# Super-enhancer function and its application in cancer targeted therapy

**DOI:** 10.1038/s41698-020-0108-z

**Published:** 2020-02-12

**Authors:** Faqing Tang, Zongbei Yang, Yuan Tan, Yuejin Li

**Affiliations:** 1 Department of Clinical Laboratory, Hunan Cancer Hospital and the Affiliated Cancer Hospital of Xiangya School of Medicine, Central South University, 410013 Changsha, China; 2 Department of Clinical Laboratory, Zhuhai People’s Hospital & Zhuhai Hospital of Jinan University, 519000 Zhuhai, China

**Keywords:** Targeted therapies, Targeted therapies, Cancer, Molecular medicine

## Abstract

Recently, super-enhancers (SEs) have been identified as a unique type of transcriptional regulation involved in cancer development. SEs exhibit a size, high transcription factor density, and strong binding to the transcriptional machinery compared with typical enhancers. SEs play an essential role in cell growth, differentiation, and disease initiation and progression including tumorigenesis. In particular, cancer-specific SEs have been proven to be key oncogenic drivers types of tumor cells. Furthermore, it has been confirmed that cancer-specific SEs can mediate the dysregulation of signaling pathways and promote cancer cell growth. Additionally, therapeutic strategies directly targeting SE components, for example, by disrupting SE structure or inhibiting SE cofactors, have shown a good curative effect on various cancers.

## Introduction

In eukaryotic cells, transcription begins with RNA polymerase binding to the promoters of DNA molecules, and transcription is regulated by transcription factors (TFs) through binding to specific DNA sequences to recruit RNA polymerase II initiation or elongation factors.^1^ The promoter region harbors transcription initiation sites. Additionally, there are some DNA sequences located near or far from promoter regions that contain multiple transcription factor binding sites. These DNA sequences are referred to as “enhancers”, and they increase gene expression.^2,3^ Recently, with the development of high throughput sequencing, increasing numbers of enhancers have been detected on a genome-wide scale. A coactivator that was proven to be an enhancer increases target gene expression.^4^ The coactivator does not bind DNA, but it pairs with TFs, to further activate gene transcription. The coactivator has general activation domains that facilitate its interaction with the basal transcription or chromatin remodeling machinery.^5^ Additionally, the histone modification of H3K4 trimethylation has been found to be associated with active promoters.^6^ DNase hypersensitive sites partially overlap with enhancer regions, and enhancer activation coincides with the DNase I hypersensitivity of these regions, which is often associated with specific posttranslational modifications of adjacent nucleosomes.^7–10^ Direct interaction or looping between enhancers and promoters has been observed and might be critical for enhancer function.^11,12^ Super-enhancers (SEs) defined as clusters of enhancers in close genomic proximity are flanked by CTCF (CCCTC-binding factor) binding sites, and are involved in regulating the expression of key genes.^13^

Gene transcriptional dysregulation, which is one of the core tenets of cancer development,^14^ involves in noncoding regulatory elements, including TFs, promoters, enhancers, SEs, and RNA polymerase II (Pol II).^15^ In particular, SEs have been found to play core roles in promoting oncogenic transcription to accelerate cancer progression.^16,17^ Herein, we introduce the concept, function and identification of SEs, and summarize the contribution of oncogenic SEs to cancer and the challenges of SEs in therapy. This will assist us to in achieving a profound understanding of the functional mechanism by which SEs regulate target gene expression.

## SEs definition and gene transcript concept

**Fig. 1:**
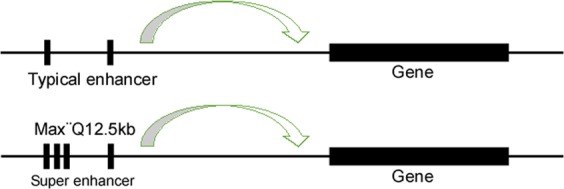
Schematic structure of typical enhancer and super-enhancer.

**Fig. 2:**
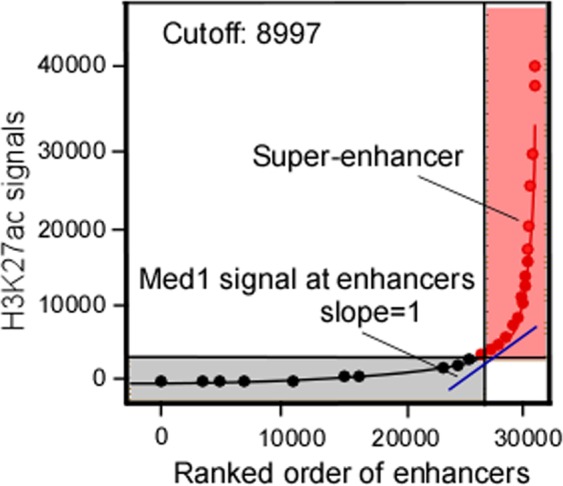
H3K27ac signal within 12.5 kb window at significant peak were ranked for enhancers. Peak with four times higher H3K27ac signals than the rest of the peak were assigned as super-enhancer (SE).

TFs are proteins that bind DNA helices at specific regulatory sequences to activate or inhibit gene transcription through a trans-activation or trans-repression domain. TFs locate their target sequences and unlock the pathway for subsequent functions, such as transcription, replication, and repair.^18^ TFs bind to and activate enhancers to initiate gene transcription.^8,19^ Enhancers are defined as short (~100–1000 bp) noncoding DNA sequences, composed of concentrated clusters of TF recognition motifs. Enhancers drive gene transcription independent of their distance, location or orientation relative to their cognate promoter.^19,20^ SEs are a large cluster of transcriptional enhancers that have been proposed to consist of a long genomic domain composed of an enhancer cluster occupied by high levels of H3K4me1, H3K27ac, p300 or master TFs. SEs span a range of more than 20 kb on average. They differ from classical enhancers in their size, transcription factor density and content, sensitivity of binding to perturbation, and active transcription (Fig. 1). SEs produce higher levels of enhancer RNAs (eRNAs) than enhancers,^13^ and present high potential to activate the transcription of target genes and drive the expression of genes.^21^ SEs are defined as follows: the identification of SEs is based on the differences in their ability to bind markers of promoter transcriptional activity, including cofactors (such as mediators and cohesins), histone modification markers (such as H3K27ac and H3K4me1), and chromatin modification molecules (such as p300) (Fig. 2).

Recently, “stretch-enhancers” were similar to SEs; stretch-enhancers induce high expression levels of target genes.^13,21–23^ A stretch-enhancer is a region of ≥3 kb according to the ChromHMM algorithm, that displays an enhancer chromatin state.^23,24^ SE’ regions are identified by binding with mediator protein (MED) 1, the enrichment of histone H3K27 acetylation (H3K27ac), or binding with cell-type-specific TFs.^25^ SEs are predicted to be located in bioinformatic stitching regions within 12.5 kb in the linear genome. As a result, SEs are longer than traditional enhancers, and often contain more than one separate region that is bound by multiple TFs.^13,21,22^

There are still some controversies about SE characterization. Since SEs were initially characterized by Whyte and Loven in 2013,^13,22^ a number of publications have referred to “super-enhancers”, although SE identification has been inconsistent. In some reports, the definition of “super-enhancer” deviates from specific properties of SEs such as chromatin marks and cofactors bound to these regions. In other studies, enhancers and SEs are not defined functionally, and enhancer stitching is an algorithmic step, not a selection criterion. The only defining feature of a “super-enhancer” is an exceptionally high degree of enrichment of transcriptional activators or chromatin marks, as determined by chromatin immunoprecipitation sequencing (ChIP-seq).^26^ Use of the term “enhancer” has also shifted from a functional definition of a DNA element that activates gene transcription to a looser definition based on chromatin profiles, characterized by DNAse I hypersensitivity, p300 binding, or H3K4me, or H3K27ac marks. The recent explosion of large-scale genomic data has led many researchers to carefully revisit these concepts.

## SE identification

For identification of SEs in mouse embryonic stem cells (mESCs), the active enhancer site was identified, and the binding sites of key TFs, Oct4, Sox2 and Nanog (OSN) binding sites were then sequenced via ChIP-seq analysis^13,21^; “constituent” enhancers were identified as a class of regulatory regions with unusually strong enrichment, all of which were merged into a large region (Fig. 1). Thereafter, the stitched enhancers were ranked by the normalized level of the Med1 signal in the genomic region, and the curve of the signal to rank plot was generated. A line with a slope of 1 tangent to the curve was used as a cutoff point to separate SEs and typical enhancers (TP), where a position above the line indicated an SE, and a position below the line indicated a TP (Fig. 2). Third, mESC enhancers were divided into two classes based on mediator levels, with one class encompassing the vast majority of enhancers, and the other containing 231 large enhancer domains. Approximately 40% of the Med1-binding signal was found to localize to SEs spanning DNA regions whose median length was an order of magnitude larger than that of a typical enhancer. The large enhancers constituted less than 3% of the total enhancer regions across the genome in terms of region and the density of binding.^13^ Some reports have shown that the co-occupancy of ESC genomic sites by OSN TFs is highly predictive of enhancer activity.^4,27^

A total of 8794 enhancers were identified in ESCs by using ChIP-seq data sets. In the differentiated cells, lineage-specific master regulators were used to generate binding plots of the OSN master TFs.^13^ SEs in mESCs were identified using key TFs such as PU.1, MyoD, T-bet, and C/EBPα from myotube cells, pro-B cells, Th cells and macrophages, and master TFs in these cells were found to be associated with SEs.^13^ SEs have been defined in many cells and tissues by tissue-specific master TFs; however, the master TFs constituting SEs are still not known, and genome-wide binding data are still limited. The various surrogate marks of typical enhancers include H3K27ac, H3K4me1, p300 and DNase, and the histone H3K27ac modification is superior to the others in mESC.^4,6,28–31^ A catalog of super-enhancers has been generated for 86 human cell and tissue types, and these SEs have been associated with genes based on cell-type-specific TFs. Thus, candidate master TFs have been identified in many cell types, which has proven to be useful for better understanding the transcriptional control of the cell state and reprogramming.^21^ SEs and their potential applications are increasingly being recognized and cited, and these findings have been widely used for the exploration of disease mechanisms, such as those of tumors.^22,32–35^ Additionally, human H1 embryonic stem (H1ES) cells were found to contain 6426 stretch-enhancers (with a mean size of 4434 bp)^23^ and 684 SEs.^21^ Among the 684 SEs, 505 overlap with a stretch-enhancer. According to the common definitions of SEs, the number of stretch-enhancers exceeds that of SEs by an order of magnitude. Thus, SEs are a subset of stretch-enhancers, and the two entities are not interchangeable as defined.

## SE structure and function

### Functional characteristics of SEs

Compared with other enhancers, SEs exhibit stronger transcriptional activation and a stronger regulatory ability for the genes that they control.^13^ Some researchers have cloned DNA fragments from SE elements into luciferase reporter vector, and then transfected the vector into ESCs to determine SE functionality. Constituent enhancer segments within SEs are defined as 600–1400 bp regions with a single peak of OSN occupancy, that generate higher luciferase activity than typical enhancer segments (3.8-fold high). SEs show a stronger ability to drive target gene transcription than typical enhancers.^36^ Interestingly, the functional interactions of SE constituents are neither superimposed nor synergistic, indicating that one component has complex effects on the activity of another component. Some SEs show increased effects on transcriptional activity, and some have inhibitory effects.^36^ SEs exhibit complicated interdependence on each other’s activity, following optimal transcriptional activity. SEs also present master transcription factor-dependent characteristics, exhibiting cell-type-specific functions and producing strong responses. In ESCs, a reduction in Oct4 leads to the loss of ESC-specific gene expression and differentiation. If SE-associated genes are more sensitive to the loss of master TFs than other genes, a reduction in Oct4 level should cause preferential loss of SE-associated gene expression. Upon knockdown of the transcription factor Oct4 in mESCs, the cells lose their pluripotent state.^13^ In this process, the expression of SE-correlated genes is decreased, and their expression is lower than that of typical enhancer related genes. These results indicate that SEs exhibit a higher interference sensitivity than TEs.^11,13,22,37^ When mediator levels are reduced using small hairpin RNAs (shRNAs), the greatest effects on gene expression were observed for SE-associated genes.^13,37,38^ If SE-associated genes are more sensitive to coactivator loss than other genes, a reduction in the levels of mediator subunits should preferentially affect the expression of SE-associated genes. Reduced levels of mediators and cohesins have the same effect on the key characteristics of the ES cell state as the loss of Oct4 itself.^38^

### Structural characteristics of SEs

**Fig. 3:**
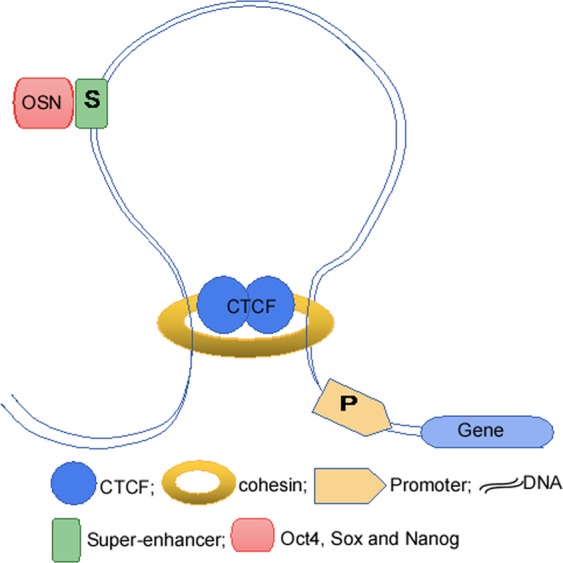
Schematic illustration of super-enhancer-mediated gene expression. Cohesin-mediated substructure of gene loops, cohesin-associated enhancer−promoter loop and cohesin-associated CTCF loop regulates gene expression. CTCF CCCTC binding factor, P promoter, OSN Oct4, Sox and Nanog.

SEs drive the expression of cell-specific genes, and are densely occupied by the transcription apparatus and its cofactors including cohesins.^11,30^ The cohesin-mediated substructure of gene loops, such as cohesin-associated enhancer−promoter loops and cohesin-associated CTCF loops, regulates gene expression (Fig. 3). The analysis of high-confidence cohesin ChIA-PET (Chromatin Interaction Analysis using Paired End Tag sequencing, ChIA-PET) interaction data revealed striking features common to loci containing SEs and their associated genes. These features consist of SEs and the associated genes located within a loop, which are connected by two interacting CTCF sites co-occupied by cohesin. A total of 84% of ESC-SEs are contained within these structures, whereas 48% of typical enhancers occur within comparable loops between two CTCF sites.^39^ Usually, one SE only contains one domain, and SEs are restricted to activate genes in SDs (Fig. 3). The loss of this restriction is likely to result in inappropriate activation of neighboring genes, which are incorrectly targeted frequently enough to cause tumors to occur.^39,40^ In mESC, SEs were defined as clusters of enhancers occupied by OSN and other mediators; in addition, CHIP-seq analysis of the terminal TFs of the Wnt, TGF and LIF signaling pathways showed that SEs bound to individual enhancers with a similar pattern to OSN.^36,41,42^ SEs can determine cell identity as a platform for dense transcription factor binding associated with different signaling pathways.^36^ eRNAs are a type of noncoding RNAs (ncRNAs) that are transcribed and synthesized from the enhancer regions of genome. eRNA plays a stimulatory role in the transcription of related genes, and exhibits a genome-wide character.^43^ eRNAs serve as a “linker” between SEs and their target genes. The coordinated regulation of transcripts by eRNA can be plotted in active RNA Pol II global nuclear run-on sequencing (GRO-seq) experiments. The transcriptional and synthetic eRNAs of enhancers are conducive to the function of enhancers, and their transcriptional levels are correlated with high activity of enhancer.^44,45^ eRNA has been studied in various cell types including neurons, macrophages, T cells and cancer cells,^44,46–48^ and approximately 30.6% of typical enhancers and 93.3% of SEs overlap with eRNAs in intergenic regions. In response to Toll-like receptor signaling in macrophages, SE-related genes and eRNA show coordinated changes. However, these signal-dependent SEs, which are associated with innate immunity and inflammation, are different from the ones identified on the basis of cellular characteristics.^45^

## SE’ functions in cancer development and applications in cancer therapy

### Oncogenic SEs activate oncogenic signaling

Compared with TEs, SEs are enriched in one or more binding sites for the terminal TFs of signaling pathways and signaling modules, and function in the maintenance of stemness and pluripotency. Extending this principle, oncogenic SEs are rich in TF binding sites, and associated with specific signaling pathways upon which cancer cells depend.^36^ Oncogenic SEs promote tumorigenesis and malignancy by upregulating oncogene transcription.^21^ Mechanistically, oncogenic SEs may activate the MAPK signaling pathway to inhibit apoptosis and promote the proliferation of cancer cells.^49^ SEs also induce overexpression of the *v-ets erythroblastosis virus E26 oncogene homolog* (*ERG*), leading to the overexpression of target genes driving the development of cancer.^50^ Additionally, oncogenic SEs upregulate *CYP24A1*, *GJA5*, *SLAMF7*, and *ETV1* in squamous cell carcinoma.^51^ The translocation of SEs upregulates *MYB* expression in adenoid cystic carcinoma (ACC), and SEs promote the expression of *TERT* in pheochromocytomas and paragangliomas.^52^

Cancer cells frequently acquire SEs to promote oncogene expression, which mediates the dysregulation of signaling pathways.^21,33,35,53^ Colorectal cancer (CRC) is driven by various oncogenic pathways,^14^ and CRC-associated SEs are enriched in transcription factor 4 (TCF4) binding sites.^36^ CRC-associated SEs are found in CRC cells but not in normal colon cells, indicating that these SEs are specific to CRC tumorigenesis. The analysis of the ChIP-seq binding profile of CRC cells shows that TCF4 is a terminal TF of the Wnt pathway and occupies at the *c-MYC* locus (Table 1). TCF4 is a well-established target of Wnt signaling that shows strong H3K27Ac signal after cancer cells acquire oncogenic SEs.^36^ Acquired SE-associated genes are enriched after the stimulation or blockage of the Wnt pathway, but not all SE genes display this response. This observation supports the notion that the acquired SEs may be dominated by TCF4 and may respond to perturbation of the oncogenic Wnt pathway.^36^ H3K27Ac ChIP-seq analysis of estrogen receptor (ESR)-positive McF-7 cells indicate that the SE of the *ESR1* gene only encodes estrogen receptor alpha (ERa) in tumor cells. Furthermore, oncogenic transcription can distinguish cancer subtypes relying on distinct signaling pathways (Table 1). In ER-positive breast cancer cells, SE-associated genes are enriched for ERa binding, whereas in triple-negative breast cancer cells, SE enriched sites are different from those of oncogenic TFs.^21,54^ One of the general functions of SEs may be to channel oncogenic signaling pathways into gene expression programs, which is required for sustaining cancer development.^36^

**Table 1:** SEs involved in cancer.

TF binding with SE	Target molecular/signal pathway	Tumor type	Tumorigenesis	Therapy	Ref.
TCF4	c-MYC/Wnt	Colorectal cancer	√		^35^
ESR	ERA/Channel oncogenic signal pathway	Breast cancer	√		^20,51^
BRD4	C-MYC	Unascertained	√	√	^21,57–60^
TAL1	MYB	Leukemia	√	√	^34^
GATA2	EVI1	AML	√		^68,70,71^
GATA3	LMO1	Neuroblastoma	√		^33^
NFE2L2, CEBPB	MYC	Lung adenocarcinoma	√		^72^
MYB/TGFBR3	MYB/RAD51B	Adenold cystic carcinoma	√	√	^73^

*AML* acute myeloid leukemia, *BRD4* bromodomain-containing protein 4, *ERA* estrogen receptor alpha, *ESR* estrogen receptor, *EVI1* ecotropic virus integration site-1, *SE* super-enhancer, *TAL1* T-cell acute lymphoblastic leukemia transcription factor 1, *TCF4* transcription factor 4, *TF* transcription factor, *TGFBR3* transforming growth factor beta receptor III.

### Chromatin regulators are regulated by an SE inhibitor

SEs were found to be associated with tumorigenesis in a myeloma cell line by Loven et al.^22^ Chromatin regulators are attractive therapeutic targets for cancer due to their deregulation in numerous cancers,^55,56^ and are regulated by an SE small-molecule inhibitor.^57,58^ The inhibition of some chromatin regulators has been proven to be efficacious for cancer treatment.^59^ Many reports focus on cancer treatment through inhibiting the expression of chromatin regulators, and inhibitors of chromatin regulators have been used to selectively inhibit the transcription of key oncogenic drivers in multiple ways. Most chromatin regulators are expressed in a broad range of normal cells, and exhibit an adverse effect on global gene expression. The small molecule JQ1 (BET bromodomain inhibitor) can selectively repress *MYC* expression by decreasing bromodomain-containing protein 4 (BRD4) binding to c-MYC SE regions.^22,60–63^ BRD4 is a member of the bromodomain and extraterminal (BET) subfamily of human bromodomain proteins, which is associated with acetylated chromatin and involved in transcriptional activation.^64,65^ It recruits positive transcription elongation factor b (P-TEFb) to regulate transcriptional elongation by RNA Pol II.^66,67^ BRD4 displays similar binding patterns to mediators, localizing to regulatory regions of the actively transcribed genes, especially at SEs. Its inhibition mediates the preferential loss of BRD4, which results in a corresponding decrease in MED1 (mediator of RNA polymerase II transcription subunit 1) binding and transcription (Table 1). Cancer cells “acquire” specific SEs near oncogenes, which occurs in a gene desert near *c-MYC* but is absent in healthy cells. This acquisition of specific SEs is thought to contribute to tumorigenesis. The key oncogenic drivers of tumor cells are regulated by SEs, which can confer disproportionate sensitivity to BRD4 coactivator loss and cause selective inhibition of transcription.^21^ This functional characteristic of cancer cell SEs may be used to identify key oncogenes and develop target drugs.^22,68,69^

### Mechanisms of oncogenic SE formation

Cancer cells acquire cancer-specific SEs that are not present in their normal counterparts. DNA translocation, transcription factor overexpression, and focal amplification frequently occur in cancer, and these changes may result from cancer cells acquiring SEs. Overexpression of *TAL1* (T-cell acute lymphoblastic leukemia transcription factor, TAL1) in a subset of acute lymphoblastic leukemia (ALL) is associated with SE formation. TAL1 upstream of an SE contains a short heterozygous somatic mutation that creates one SE and introduces binding motifs for the MYB transcription factor in noncoding sites^35^ (Table 1). MYB binding with the SE generates a positive feedback loop that reinforces its own expression, which activates an MYB-dependent oncogenic transcriptional program^40,53,70,71^ (Table 1).

Insertion mutations, chromosomal inversions and translocations play a central role in the pathogenesis of almost all cancers. A distal enhancer of the *GATA2* gene arising upon chromosomal 3q rearrangement ectopically activates EVI1 (ecotropic virus integration site-1) expression, which leads to the concomitant loss of *GATA2* transcription.^40^ The loss of *GATA2* is associated with acute myeloid leukemia (AML), and *GATA2* haploid in sufficiency might provide the precise context for EVI1-mediated oncogenic transformation^40,72,73^ (Table 1). In addition to the above cases, single nucleotide polymorphisms also have a direct effect on the regulation of oncogenic SEs. In neuroblastoma, GATA3 plays a central role in regulating *LMO1* expression. SE formation at the LMO1 oncogene depends on GATA protein3 binding at the conserved intronic GATA. Knockdown of GATA3 results in a decrease in LMO1 and suppression of cell growth. The protective T allele (TATA) disrupts GATA3 binding, and leads to reduced recruitment of H3K27Ac, which is negatively associated with the LMO1 SE in neuroblastoma cells^34^ (Table 1). Focal amplification of SEs can also result in aberrant oncogene expression. In lung adenocarcinoma, the focal amplification of ~450 kb downstream of the *MYC* locus leads to SE formation, and drives high expression of the oncogene, in which NFE2L2 and CEBPB are necessary to maintain SE activity.^74^ A chromosomal translocation in ACC repositions an unrelated SE in proximity to the *MYB* oncogene, resulting in high *MYB* expression^75^ (Table 1).

### Therapeutic strategy of targeting oncogenic SEs

Since cancer cells exhibit increases in the level of oncogenic transcriptional activity and growth-promoting pathways, a specific therapeutic strategy targeting oncogenic transcription has been developed.^16,76^ Inhibiting oncogenic transcription is an attractive therapeutic option; however, transcription is a biological process that is fundamental to all living cells, and inhibiting transcription has dire consequences for cell gene expression^16,77^; thus, any clinically useful transcriptional inhibitor should selectively target oncogenic transcription with only minimal toxicity in normal cells. JQ1, iBET, and bromodomain inhibitors selectively bind to the domains of BRD4,^78,79^ which causes selective repression of the *MYC* oncogene in a wide range of tumors including multiple myeloma (MM), Burkitt’s lymphoma (BL), AML, and ALL^60–63,80^ (Table 2). SE-associated transcription depends on the cooperative binding of BRD4 and mediators, and the recruitment of the CDK7-containing initiation complex (TFIIH) and CDK9-containing elongation complex (p-TEFb). JQ1 is a competitive inhibitor of BRD4 that works by reducing BRD4 occupancy and reducing levels of MED1 binding, resulting in Pol II stalling and impaired elongation. Such an SE-driven transcriptional program is mainly dependent on BRD4; it is important to maintain oncogenic identity and pluripotency.^76,81^ iBET, which is similar to JQ1, has also been applied for cancer treatment.^82^ In addition to BET inhibitors, targeting CDKs to regulate RNAPII initiation and elongation shows great potential^83^ (Table 2).

**Table 2:** Therapeutic SEs and inhibitors.

SE inhibitor	Molecular target	Function	Tumor type	Ref.
JQ1	BRD4	Reducing BRD4 occupancy and MED1 binding, Pol II stalling and elongation impairment	AML	^74,79^
IBET	CDKs	Regulating RNAPII initiation and elongation	Unascertained	^81^
THZ1	CDK7	Inhibiting phosphorylation of CTD of RNA Pol II and hindering promoter proximal pausing	Neuroblastoma	^65^
	MYCN	Repression of MYCN-dependent transcriptional amplification	Neuroblastoma	^65^

*AML* acute myeloid leukemia, *BRD4* bromodomain-containing protein 4, *CDK* cyclin-dependent kinase, *CTD* carboxyl-terminal domain, *Pol II* polymerase II.

Recent studies have demonstrated that covalent inhibitors of CDK7 and 12 electively kill cancer cells by inhibiting SE-driven oncogenic transcription, but lack systemic toxic effects in vivo.^76,81^ THZ1 is a highly specific covalent inhibitor of CDK7, and CDK7 is known to promote transcription activation through phosphorylation of RNA Pol II; THZ1 inhibits the phosphorylation of the carboxyl-terminal domain (CTD) of RNA Pol II and hinders promoter proximal pausing.^84^ Because SEs are enriched at the paused RNA Pol II, THZ1-induced deficiency at the pause sites leads to the decreased occupancy of Pol II at these enhancers, culminating in transcriptional inhibition^85,86^ (Table 2). The enrichment of master TFs in SEs maintains gene expression through autoregulatory feed-forward loops, and their depletion may lead to the inhibition of transcriptional output. The MYC oncoprotein has been verified to stimulate tumor cell growth and proliferation through the amplification of gene transcription; therefore, inhibiting MYC function might be an attractive therapeutic option. It has been demonstrated that genomic amplification of the *MYCN* oncogene by promoting SEs causes upregulation of the active transcriptional program of neuroblastoma (NB) cells and sensitizes NB cells to the inhibition of CDK7. When a covalent inhibitor of CDK7 is used to disrupt the transcription of amplified *MYCN* in NB cells, the oncoproteins are downregulated with the consequent strong suppression of *MYCN*. THZ1 selectively targets *MYCN*-amplified NB cells, and THZ1 target treatment leads to the preferential downregulation of SE-associated genes and significantly represses MYCN-dependent transcriptional amplification^68^ (Table 2). The requirement of tumor cell for the high expression of oncogenes contributes to their vulnerability to super-enhancer disruption. Some SE inhibitors cause preferential loss of SE-regulated elements and TFs at SE-associated genes, for example BRD4 inhibition leads to the preferential disruption of SEs. SEs occupied by BRD4 regulate critical oncogene expression in MM, which shows that BRD4 inhibition leads to preferential disruption of these super-enhancers. This preferential disruption of SE function may be a general approach for selectively inhibiting the oncogenic drivers of cancer cells.^22^

## Conclusion

SEs are large clusters of transcriptional enhancers that drive gene expression to control cell identity. Compared with normal enhancers, SEs display a unique structure and strong functional properties. Although there is still a lack of uniform rules for the definition of SEs, the differences in transcription factor density and content, binding sensitivity, and active transcription are used to distinguish SEs from ordinary enhancer,^13^ and whether this can become a comprehensive concept remains to be further studied and verified.^26^ SEs have been proven to be valuable in pathologic studies of disease. SEs are key regulators of the expression of key oncogenes in many tumor cells. Some reports have shown that SEs can promote oncogene overexpression, and disrupting SE structure and inhibiting cofactors may be specific routes for cancer therapy. Recent studies have demonstrated that some inhibitors (such as JQ1 and CDK7) selectively kill cancer cells by inhibiting SE-driven oncogene transcription. However, transcription is a fundamental biological process shared by all living cells, and targeting transcription may therefore have dire consequences for global gene expression. Along with the development of SE research, there may be novel ideas about therapeutic techniques for diseases such as cancer and other complex diseases.
